# Ten simple rules for getting started on Twitter as a scientist

**DOI:** 10.1371/journal.pcbi.1007513

**Published:** 2020-02-10

**Authors:** Veronika Cheplygina, Felienne Hermans, Casper Albers, Natalia Bielczyk, Ionica Smeets

**Affiliations:** 1 Department of Biomedical Engineering, Eindhoven University of Technology, Eindhoven, The Netherlands; 2 Leiden Institute of Advanced Computer Science, Leiden University, Leiden, The Netherlands; 3 Software Engineering Research Group, Delft University of Technology, Delft, The Netherlands; 4 Heymans Institute for Psychological Research, University of Groningen, Groningen, The Netherlands; 5 Stichting Solaris Onderzoek en Ontwikkeling, Nijmegen, the Netherlands; 6 Science Communication and Society, Institute of Biology, Leiden University, Leiden, The Netherlands; Dassault Systemes BIOVIA, UNITED STATES

## Introduction

Twitter is one of the most popular social media platforms, with over 320 million active users as of February 2019. Twitter users can enjoy free content delivered by other users whom they actively decide to follow. However, unlike in other areas where Twitter is used passively (e.g., to follow influential figures and/or information agencies), in science it can be used in a much more active, collaborative way: to ask for advice, to form new bonds and scientific collaborations, to announce jobs and find employees, to find new mentors and jobs. This is particularly important in the early stages of a scientific career, during which lack of collaboration or delayed access to information can have the most impact.

For these reasons, using Twitter appropriately [1] can be more than just a social media activity; it can be a real career incubator in which researchers can develop their professional circles, launch new research projects and get helped by the community at various stages of the projects. Twitter is a tool that facilitates decentralization in science; you are able to present yourself to the community, to develop your personal brand, to set up a dialogue with people inside and outside your research field and to create or join professional environment in your field without mediators such as your direct boss.

This article is written by a group of researchers who have a strong feeling that they have personally benefited from using Twitter, both research-wise and network-wise. We (@DrVeronikaCH, @Felienne, @CaAl, @nbielczyk_neuro, @ionicasmeets) share our personal experience and advice in the form of ten simple rules, and we hope that this material will help a number of researchers who are planning to start their journey on Twitter to take their first steps and advance their careers using Twitter.

## Twitter terms

Before we start with the rules, inspired by [2] we briefly introduce a number of relevant Twitter terms.

- Timeline—The tweets from the people you follow.
- Retweet (RT)—sharing a tweet that was originally made by someone else.
- Quote-tweet—sharing a tweet by someone else in a quote, while adding your own comments.
- Like (♡), used for showing you like a tweet—a fast way to give feedback without replying. There is no similar function for disliking a tweet.
- Notifications—Tweets that mention you and replies, retweets and likes for your tweets.
- Mentioning (@)—If you mention someone with their handle (“This paper by @CaAl is great”), your tweet will show up in their notifications.
- Direct message (DM)—A private message that is only visible to the sender and the specifically identified recipients. By default, regular Twitter messages are visible to the whole world, including (via search engines such as Google) people who do not have a Twitter account.
- Hashtag (#)—used to make it easier to find tweets with a common theme by defining ad hoc keywords, for instance tweets about a conference (#ICA19) or career talks (#PhDChat).
- List—a list of Twitter users that can be public (followed by anyone) or private. Lists can be used to follow accounts that tweet about specific topics, but which you don’t want to follow yourself.
- Hat Tip or Heard Through (HT), used for thanking the source of a tweet.
- Subtweeting—Tweeting about somebody without explicitly mentioning their handle, so that they are not informed of your comment (see ‘Mentioning’).
- Live-tweeting—Tweeting short summaries of an event, for example of a conference talk, as it is happening.
- Thread—A series of tweets on one subject, for instance ten tweets about a new research paper.

## Rule 1: Start somewhere, but show up

To get into the habit of using Twitter, you need to do just that: start. You can do this even before you finish reading this article! You don’t need to know everything about Twitter before posting a tweet, just like you don’t need to know everything about running a marathon before going for a jog. Create an account (you can change your username later) and tweet something—for example, a comment about a paper you are writing or a conference you are going to. This way you can use ideas that you already have—but put them into the form of a tweet, which aligns with the advice of not treating research and outreach as separate entities [1].

**Fig 1:**
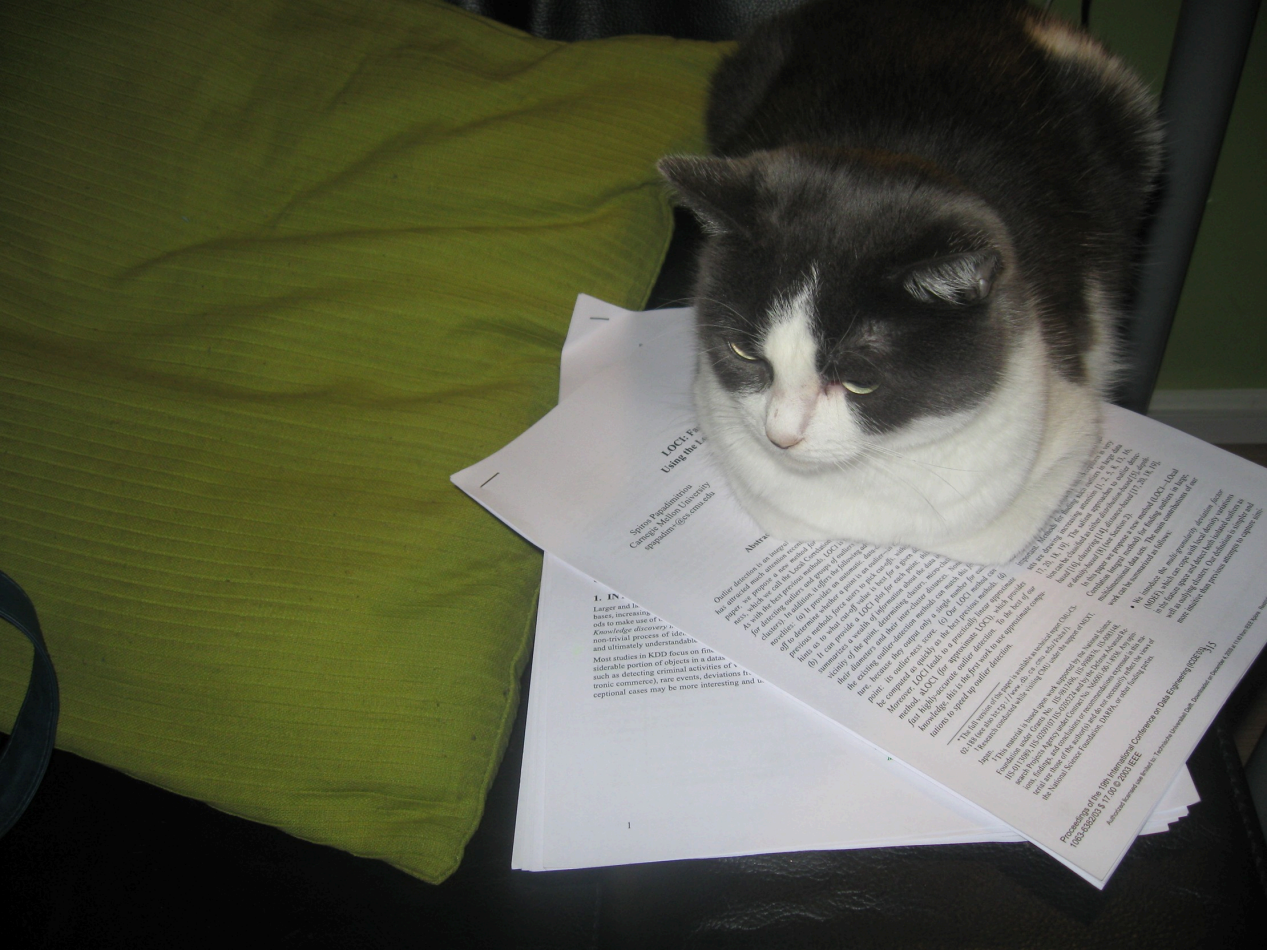
Cats. Pictures that someone might post on Twitter to convey they are reading papers AND like cats. Possible relevant hashtags include #AcademicsWithCats, #Caturday, #ECRchat (chat as in conversation, not as in cat in French).

A bigger challenge is to continue tweeting regularly once you have started, which is essential to habit change [3], similar to going running every few days. There is no one-size-fits-all set of rules for this, but we provide a few suggestions here. Once that paper is completed or the conference is over, perhaps you can follow other people in your field and respond to their tweets first, or check hashtags where everybody is invited to contribute, such as #PhDChat or #ECRChat. Such general hashtags are also great ways to discover and follow people who you would not run into at your department or conferences. Hashtags are good places to start posting your own questions or content—for an easy start, try #AcademicsWithCats, where you can show off your cat helping you with reviewing duties (Fig 1).

## Rule 2: Discover opportunities in academia

To Early Career Researchers (ECRs), Twitter has become an invaluable source of information [4,5]. One can follow granting agencies, particular labs and dedicated career columns in popular research journals, which allows for tracking the first-hand information on recently opened positions, grant calls, and new trends on the academic job market.

But even more importantly, Twitter culture has exposed a part of academia that has traditionally always been hidden from view, namely the inception of new research activities. Now, ECRs can observe and even join the process of creating national or international research projects (for instance, [6] stems from a discussion at Twitter and Bik et al [1] write their work resulted from online interactions). Senior researchers openly share ideas through Twitter and this can lead to the development of new concepts which often move on to become fully-fledged research projects.

Many researchers active on Twitter are also open about their everyday struggles, share their frustrations from rejections and bureaucracy, and support each other emotionally [7]. This is especially valuable for ECRs who have experienced the black side of academic life: patronizing behaviors, inequality and lack of inclusivity in research projects. For these researchers, Twitter gives an opportunity to follow senior role models who have managed to develop successful research careers and are willing to openly share their difficult experiences from the past. Twitter can play the role of a distributed mentoring system which can have a profound impact on a researcher’s self-image and can help to mitigate imposter syndrome. Similar reasons are listed by [8], who crowdsourced over 400 responses from academics on why they use Twitter.

For ECRs, starting Twitter activity may be hard. Thus, we recommend joining a peer group, together with members of your local research group, together with other collaborators or friends in the research community. In this way, you can make sure that you have a few followers right at the start, and that your peer group helps you in distributing your content.

## Rule 3: Tweet stuff

Twitter is useful for absorbing and sending information, but the potential for interaction with fellow scientists is where its real power lies [9]. Especially for new Twitter users, it is good practice to retweet other users’ content. Retweeting means sharing someone else’s tweet with your followers [10]. This way, your timeline demonstrates the scientific topics of your interest. You can either retweet directly or quote-tweet, which means adding your own commentary to the tweet. Quote-tweeting adds a personal flavor to your tweets.

Interact with others by asking them their opinion on your (re)tweet. Interactivity can be increased by using (simple) polls. Do not hesitate to ask scientists questions about their work (e.g., “can you share the code for that paper?”): they signed up to Twitter because they intend to interact. Also, feel free to ask questions, both content-related (“Which *R* package can we use for this?”) and logistic (“How do you handle requests for letters of recommendation?”).

Many scientists are bilingual on Twitter, tweeting both in their mother tongue and in English. Do not worry that this might be confusing: Twitter users can easily skip over tweets in languages they are not proficient in, or make use of the automatic translation facility that is offered on every tweet in a foreign language.

## Rule 4: Learn the rules

It should go without saying, but as with any situation where you communicate with others, you should treat them with respect. If you wouldn’t do something in real life, you probably should not do it on Twitter. Next to conventional politeness, there is also some basic Twitter-etiquette that you should follow: credit ideas from others by mentioning them with HT (there is some discussion whether HT means “hat tip” or “heard through”—but there is absolutely no discussion about whether to use it) and their username. Be careful about subtweeting people (i.e., talking about them or their work without mentioning them explicitly in your tweet). It is like gossiping, but then clearly visible to everyone. This is especially problematic if you are in a position of privilege with respect to the person you are quoting. Repeatedly tweeting at somebody who is not interested is another behavior to avoid. This is called “sealioning”, and is one of the negative sides of Twitter experienced at a higher rate by underrepresented groups on Twitter.

Use hashtags when appropriate. You can give tips for people who are interesting to follow on Friday with: #FollowFriday or share stories of colleagues who are helping others with #AcademicKindness. Next to hashtags, there are many memes that are like little inside jokes; use those only when you really understand them. One of our favorite examples is “Asking for a friend” used in tweets where you are asking something for yourself that is a bit embarrassing. One example is: “Why is Reviewer 2 always the one who says you should have written on a different topic using a different method? Asking for a friend” [11].

Also, do not respond impulsively if someone is critical towards your research on Twitter. Diplomacy is one of the key components to building a scientific reputation [12].

It might be good to check whether your institute has any social media guidelines (and stick to those). For example, Stanford University tells researchers not to endorse commercial partners (https://ucomm.stanford.edu/policies/social-media-guidelines/) and the University of Oxford warns employees to be mindful of what pictures might reveal in the background (https://www.admin.ox.ac.uk/personnel/during/socialmedia/). Some employers, for example Leiden University (https://www.medewerkers.universiteitleiden.nl/binaries/content/assets/ul2staff/reglementen/communicatie-en-marketing/gedragscode-social-media-2012.pdf) may ask you to add a disclaimer that opinions are your own.

## Rule 5: Take care of yourself

Unfortunately, online conversation can easily go off the rails and you might need to protect yourself from trolls and nasty discussions. Curate who you choose to follow and be prepared to mute or block people. Muting people means that you will generally not see their tweets anymore, but they will still be able to read your tweets and reply to them. You can also mute specific words for which you do not want to see tweets: names of politicians, TV shows, and many other topics. This can be useful if you want to follow a colleague who is both tweeting about interesting research and ranting about politics. When muting somebody, the person will not be aware that you muting them. Blocking is a more drastic measure than muting and makes the blocked user unable to read your tweets or react to them. The blocked user will be aware that they are blocked, if they attempt to read your tweets. There is no single guideline on when to block somebody—some may only support blocking trolls, and refrain from blocking other scientists who may be critical, while others believe it’s perfectly fine to mute or block any account [13]. A good rule of thumb might be to block somebody if they have a repeated negative effect on your Twitter experience. For example a critic with many followers who frequently quote-tweets your posts might want to start a discussion, but could end up unleashing many trolls, in which case blocking would be the only sure way to prevent the same from happening again.

Never feel pressured to read everything on Twitter. It is perfectly okay to not look at Twitter for hours or even days. You definitely don’t need to read up your entire timeline when you have been away, just join the conversation at the point where you fall in again. See it as dipping into the stream. It might be good to read up on your mentions (rather than all notifications), since these are personal remarks and questions just for you.

In fact, using Twitter irresponsibly can yield negative impact on mental health. One should remember that tweeting can be addictive and should therefore be self-managed with care. Getting notifications on social media activates the same dopamine loop as gambling [14]. When you become addicted to Twitter and you are not able to check your phone, adrenaline and cortisol are released to your body [15]. Therefore, it is beneficial to dose the amount of Twitter in your daily life: set fixed times during the day or during the week when you remain logged into your Twitter account, and switch it off otherwise. One strategy is to limit Twitter use to your time on public transport, or while in the queue to order a coffee.

## Rule 6: Build your own community

A nice feature of Twitter is its asymmetry: you do not have to follow everyone who follows you and vice versa. You can follow big names you have never met and should not be afraid to join a conversation with more senior researchers or people you do not know. Following diverse voices will broaden your horizon.

To a certain extent, you can also determine the kind of community your followers form. Twitter offers the possibility for communicating directly with people who would otherwise not find out about your work and field. This is a very relevant form of science communication, since it is a transaction of ideas and not just a transmission [16]. If this appeals to you, you could share and explain papers that might be interesting to people outside your field, comment on news items from your expertise and answer questions from people you do not know. And vice versa: posing questions is also a great way to build a community, since people who share common interests will find each other on your timeline.

## Rule 7: Interface with real life

Twitter is a great way to make networking more easy and fun and less scary. For example, it can change your experience in attending a conference [2]. Firstly, you can find interesting people to follow by browsing through the list of users who include the conference hashtag in their tweets. If a conference does not have a well-defined hashtag, try a few options to see what most people are using (for example #sigcse19 versus #sigcse2019; [2]). One way to use the conference hashtag is to announce your presence or your research ahead of time. Another possibility is to live-tweet—briefly summarize talks you are attending—which is helpful both for other people at the conference, as well as people who could not travel, thus potentially communicating the content to a more diverse audience. It also helps if your Twitter avatar includes your face, so that people who also follow the conference hashtag can spot you in the coffee breaks at the conference site.

Following people from conferences has value *after* the conference too. Twitter is a good way to stay in touch after a conference, aa it provides a low-cost way of tracking what people are working on or interested in. Knowing a bit about their interests in research and in life will make it a lot easier and less scary to say “hi” to people the year after!

This advice also holds outside of conferences: if you are approaching people you do not know, for example, to ask for a committee membership or a collaboration—you can check out their Twitter account and get an idea, not just of what they work on, but also their style of communication in general. Finally, when you are visiting a conference or university, you can advertise your visit in advance and perhaps plan extra meetings or talks.

**Fig 2:**
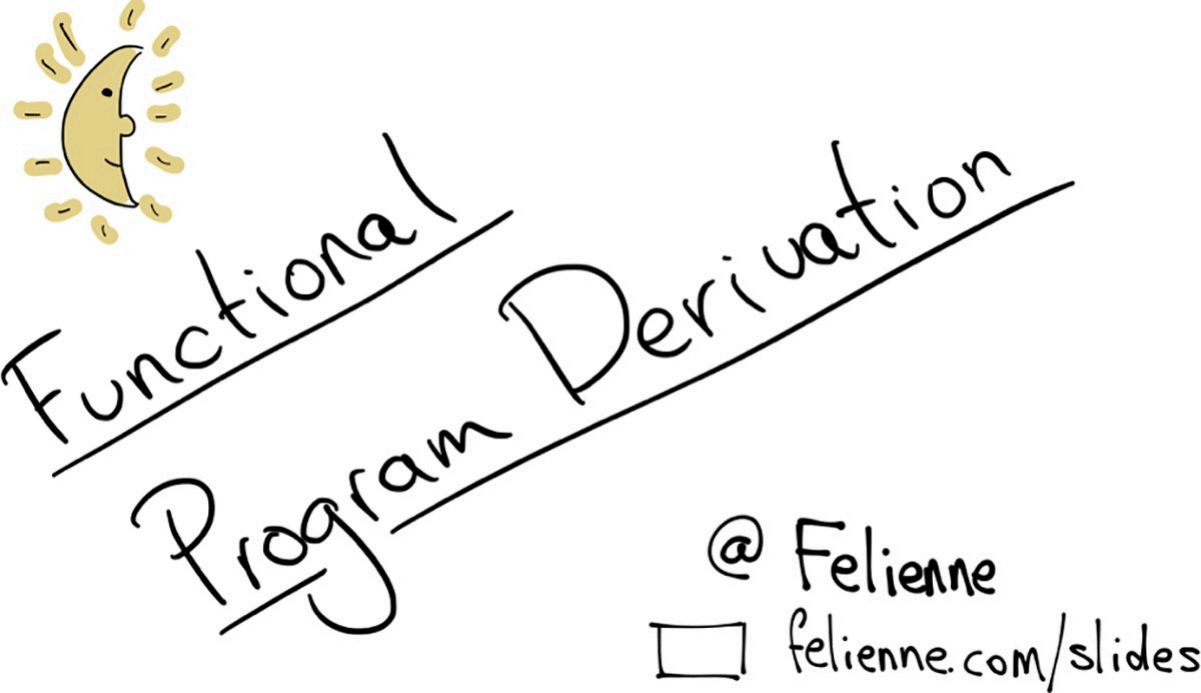
Title slide. Example of adding your Twitter handle to your slides. Source: [17].

There are also possibilities to make your Twitter network more extensive and more interesting by interfacing with real life. Put your Twitter handle on slides (see Fig 2) and other material, so people that you meet at conferences or talks can follow you, thus further extending your Twitter network.

## Rule 8: Spread your message

Whenever you have some scientific accomplishment, you can share this information by, for instance, sharing a link to a preprint or vacancy notice. In order to get the best exposure, summarize the content and include an image and an appropriate hashtag (e.g., #phdposition). You can “tag” (i.e., mention the usernames of) scientists if you think your tweet is of special interest to them, but do not overdo this. Retweeting can also be a way to put scientists whom you feel deserve more attention in the spotlight.

Adding humor to your tweets (e.g., [18, 19]) can help to get you noticed by more users who might then follow you and also receive updates of your more serious contributions. Twitter is not only useful to communicate with fellow scientists, but also with people outside of science [20]. This does, however, require a different style of communication [21].

Although Twitter is accessible worldwide, it is useful to consider the time of tweeting things, especially when your tweet has more of a local audience. Many people do not read the whole of their timeline and so may never see tweets that were sent while they were asleep. Also keep in mind that not all scientists work at weekends, so science Twitter is a bit quieter then than on weekdays. Tweets for global audiences can be retweeted at a later moment in order to reach different time zones.

## Rule 9: Be a real person

Even if you use Twitter only for professional purposes, consider opening up a little bit to show your followers you are a real person. People outside your field are not likely to follow you if your tweets are only about sharing events, articles, and positions in your own field. You need to add an extra ingredient—your opinions, or something personal—to what you share. One way to do this is through sharing failures: a rejected paper or job application, or even a spilt coffee. This is a great way to give and receive moral support from other academics.

Beyond academia, sharing something personal shows non-academics that scientists are just people too. You don’t need to go into details of your life to do this—think about a nice book you have read recently, a concert you visited, or your cat, as long as you keep the vibe positive. Similar to [22], we think that humor and individuality should not impact the perceived value of science.

## Rule 10: Great power & great responsibility

Once you reach a substantial number of followers, let’s say around 1,000, Twitter changes a bit from being a place where you go to share to a place where other people go to learn from you! This change can be a bit similar to going from PhD student to staff: suddenly you are (part of) defining what is and what isn’t appropriate, interesting or cool.

This responsibility means that you will need to spend a bit more time to think about what you tweet. There are cases of people getting in trouble for things they have shared through Twitter—going as far as being taken to court for retweeting somebody else’s opinion [23]. [8] also report an instance of having to seek help from the university’s legal service. One challenge as you acquire more followers is to strike a balance between remaining open and personal, while making sure you do not upset too many people in power, whether inside or outside your own institution or field.

On the other hand, a large Twitter network also helps you to spread important ideas, and help people learn about opportunities you know about. To a certain extent, Twitter is an equalizer, whether you are a full professor or an undergraduate student.

You can also use Twitter to lift deserving people up. Ensure that you properly credit collaborators, mentors, and especially students to increase their visibility. A great hashtag to promote researchers is #ScholarSunday, an academic version of #FollowFriday created by Dr. Raul Pacheco-Vega.

## Conclusion

In this article, we have introduced the way in which researchers can benefit from using Twitter, and we listed a number of tips for effective tweeting, both for people who are considering joining Twitter and for those who are already ardent Twitter users.

As with any other aspect of human life, Twitter has trade-offs between quantity and quality. It is, unfortunately, a frequent practice to retweet any content spread by one’s collaborators or friends without even reading it. This allows users with high numbers of followers to gain further attention with minimal effort in a kind of “snowball” effect. But chasing after numbers in this or other ways (e.g. tweeting with lots of hashtags in order to maximize your number of followers) is typically a short-sighted strategy, as Twitter is also inhabited by a large number of bots (automated accounts) which will tend to follow you and instead of contributing to your networking efforts, distract you by flooding you with a lot of unwanted content.

Twitter is also a public platform and one should be careful about spreading any content that is judgmental about other people, including their personality, their beliefs and their work. Some people have an inborn sense of diplomacy while others need to learn it the hard way. You might find it prudent to observe other researchers’ interactions on Twitter for a while before you indulge in heated conversations on any topic.

Furthermore, one should remember that Twitter’s business model is based on advertising. Therefore, the users need to be aware that some of the content is sponsored and aims solely at selling products to the user. As mentioned earlier, Twitter is also populated by bots which are designed to enable certain accounts to increase their numbers of followers. This often takes the form of a certain users following you and then unfollowing you immediately after you follow them back.

Regardless of these potential downsides, Twitter is still strongly recommended for anyone who needs to develop themselves in academia, to learn and to teach, to develop and tighten bonds with researchers overseas, and to join the #AcademicTwitter community.
